# Perceived Racism and Demographic, Mental Health, and Behavioral Characteristics Among High School Students During the COVID-19 Pandemic — Adolescent Behaviors and Experiences Survey, United States, January–June 2021

**DOI:** 10.15585/mmwr.su7103a4

**Published:** 2022-04-01

**Authors:** Jonetta J. Mpofu, Adina C. Cooper, Carmen Ashley, Sindhura Geda, R. Lee Harding, Michelle M. Johns, Adiaha Spinks-Franklin, Rashid Njai, Davia Moyse, J. Michael Underwood

**Affiliations:** ^1^Division of Adolescent and School Health, National Center for HIV, Viral Hepatitis, STD, and TB Prevention, CDC; ^2^ICF International, Rockville, Maryland; ^3^Division of Developmental Pediatrics, Department of Pediatrics, Texas Children’s Hospital/Baylor College of Medicine, Houston, Texas; ^4^Office of Minority Health and Health Equity, CDC

## Abstract

Perceived racism in school (i.e., a student’s report of being treated badly or unfairly because of their race or ethnicity) is an important yet understudied determinant of adolescent health and well-being. Knowing how perceived racism influences adolescent health can help reduce health inequities. CDC’s 2021 Adolescent Behaviors and Experiences Survey (ABES), an online survey of a probability-based, nationally representative sample of U.S. public- and private-school students in grades 9–12 (N = 7,705), was conducted during January–June 2021 to assess student behaviors during the COVID-19 pandemic. CDC analyzed data from ABES to measure perceived racism and the extent to which perceptions of racism are associated with demographic, mental health, and behavioral characteristics. Mental health and behavioral characteristics analyzed included mental health status; virtual connection with others outside of school; serious difficulty concentrating, remembering, or making decisions; and feeling close to persons at school. Demographic characteristics analyzed included sex, race and ethnicity, and grade. Prevalence of perceived racism and associations between perceived racism and demographic, mental health, and behavioral characteristics are reported overall and stratified by race and ethnicity. Approximately one third (35.6%) of U.S. high school students reported perceived racism. Perceived racism was highest among Asian (63.9%), Black (55.2%), and multiracial students (54.5%). Students who reported perceived racism had higher prevalences of poor mental health (38.1%); difficulty concentrating, remembering, or making decisions (44.1%); and not feeling close to persons at school (40.7%). Perceived racism was higher among those students who reported poor mental health than those who did not report poor mental health during the pandemic among Asian (67.9% versus 40.5%), Black (62.1% versus 38.5%), Hispanic (45.7% and 22.9%), and White students (24.5% versus 12.7%). A better understanding of how negative health outcomes are associated with student experiences of racism can guide training for staff and students to promote cultural awareness and antiracist and inclusivity interventions, which are critical for promoting safe school environments for all students.

## Introduction

Racism, defined as “a system of structuring opportunity and assigning value based on the social interpretation of how one looks (i.e., race) that unfairly disadvantages some individuals and communities, unfairly advantages other individuals and communities, and saps the strength of the whole society through the waste of human resources,” (*1*) is a critical social determinant of health and a key driver of systemic inequities in health outcomes (*1*,*2*). Racism influences the health and well-being of racial and ethnic minority persons and families throughout the lifespan and contributes to racial and ethnic disparities in health outcomes (*2*,*3*). Self-reported or perceived racial discrimination among adults is associated with poor mental health, high-risk behaviors (e.g., substance use and misuse), physical health conditions (e.g., hypertension and cardiovascular disorders), and other adverse health outcomes (*3*). Although less is known about perceptions of racial discrimination among children and adolescents (*4*,*5*), a growing body of research describes associations between racial discrimination and health outcomes for youths. Experiences of racial discrimination are associated with poor mental health (e.g., anxiety, depression, and low self-esteem), health risk behaviors, reduced social and adaptive functioning, and delinquent behaviors among youths (*6*,*7*). Racial discrimination in educational settings contributes to racial disparities in academic achievement and educational attainment, which are important markers for long-term health outcomes (*7*).

Understanding experiences of racism and racial discrimination among adolescents and how those experiences influence health is important to promote equitable health outcomes for racial and ethnic minority youths. To understand the effects of racism on health, well-defined, consistent definitions and reliable measures of racial discrimination are critical (*6*). To date, few measures have been designed to assess perceived racial discrimination among child and adolescent populations (*5*).

Throughout the COVID-19 pandemic, communities of color have been disproportionately affected by severe outcomes of COVID-19 (e.g., hospitalizations, intensive-care admissions, or in-hospital deaths) and limited access to quality health care (*8*). Structural racism, a central pathway through which racism influences health (*3*), is associated with inequities in COVID-19 morbidity, hospitalization, and mortality (*8*). Less is understood about adolescent perceptions of racism and its consequences during the COVID-19 pandemic. Perceived racism in school is an important yet understudied determinant of adolescent health and well-being, and knowing how perceived racism influences adolescent health can help reduce health inequities. In spring 2021, CDC implemented the Adolescent Behaviors and Experiences Survey (ABES) to assess student behaviors during the pandemic. ABES, a nationally representative sample of high school students, included a single-item measure of perceived racism. Using ABES data, this report examines perceived racism and the extent to which perceptions of racism are associated with behavioral health outcomes among adolescents. The findings in this report can help inform the development of school staff trainings and interventions to support the health and well-being of all students.

## Methods

### Data Source

Data from the ABES conducted by CDC during January–June 2021 were used to assess student behaviors during the COVID-19 pandemic. ABES was a one-time, probability-based online survey of U.S. high school students. ABES used a stratified, three-stage cluster sampling approach to obtain a nationally representative sample of public- and private-school students in grades 9–12 in the 50 U.S. states and the District of Columbia (N = 7,705). Participation in ABES was voluntary; each school and teacher decided whether students completed the survey during instructional time or on their own time. Additional information about ABES sampling, data collection, response rates, and processing is available in the overview report of this supplement (*9*). The ABES questionnaire, datasets, and documentation are available at https://www.cdc.gov/healthyyouth/data/abes.htm.

### Measures

Self-reported measures of lifetime perceived racism at school and four mental health and behavioral characteristics were included in this analysis (Table 1). Mental health and behavioral characteristics included mental health status during the COVID-19 pandemic; virtual connection with family, friends, and other groups outside of school; difficulty concentrating, remembering, or making decisions; and feeling close to persons at school. Demographic characteristics included sex, race and ethnicity (non-Hispanic American Indian or Alaska Native [AI/AN], non-Hispanic Asian [Asian], non-Hispanic Black [Black], Hispanic or Latino [Hispanic], non-Hispanic persons of multiple races [multiracial], non-Hispanic Native Hawaiian or other Pacific Islander [NH/OPI], and non-Hispanic White [White]), and grade (9, 10, 11, or 12).

**TABLE 1:** Variables, questions, response options, and analytic coding for perceived racism and behavioral characteristics — Adolescent Behaviors and Experiences Survey, United States, January–June 2021

Variable	Question	Response option	Analytic coding
Perceived racism*	During your life, how often have you felt that you were treated badly or unfairly in school because of your race or ethnicity?	Never, rarely, sometimes, most of the time, always	Never versus ever (rarely, sometimes, most of the time, always)
Poor mental health during the COVID-19 pandemic	During the COVID-19 pandemic, how often was your mental health not good? (Poor mental health included stress, anxiety, and depression.)	Never, rarely, sometimes, most of the time, always	Never versus ever (rarely, sometimes, most of the time, always)
Virtual connection with family, friends, or other groups outside of school during the COVID-19 pandemic	During the COVID-19 pandemic, how often were you able to spend time with family, friends, or other groups, such as clubs or religious groups, by using a computer, phone, or other device? (Do not count attending school online.)	Never, rarely, sometimes, most of the time, always	Never versus ever (rarely, sometimes, most of the time, always)
Serious difficulty concentrating, remembering, or making decisions because of a physical, mental, or emotional problem	Because of a physical, mental, or emotional problem, do you have serious difficulty concentrating, remembering, or making decisions?	Yes, no	Yes versus no
Feel close to persons at your school	Do you agree or disagree that you feel close to people at your school?	Strongly agree, agree, not sure, disagree, strongly disagree	Yes (strongly agree, agree) versus no (not sure, disagree, strongly disagree)

* See Supplementary Table at https://stacks.cdc.gov/view/cdc/115178 for full distribution of the perceived racism variable. The question was derived from the Perceptions of Racism in Children and Youth (PRaCY) scale.

### Analysis

Weighted prevalence estimates and 95% CIs for perceived racism (students who reported they were treated badly or unfairly in school because of their race or ethnicity over their lifetime) were calculated overall and by sex, race and ethnicity, grade, mental health, and behavioral characteristics (Table 2). Prevalence estimates and 95% CIs for associations between perceived racism and demographic, mental health, and behavioral characteristics also were calculated stratified by sex and race and ethnicity (Tables 3 and 4). Estimates were suppressed when n<30; consequently, NH/OPI students were only included in Table 2. Statistically significant differences in perceived racism by demographic and behavioral characteristics were determined using a two-sided chi-square test at the p value <0.05 level. Pairwise differences in perceived racism for grade were calculated by race and ethnicity and considered statistically significant if the *t*-test p value was <0.05. Analyses were completed using SUDAAN (version 11.0.1; RTI International) to account for the complex survey design and weighting.

**TABLE 2:** Percentage of high school students who reported experiencing perceived racism during their life* — Adolescent Behaviors and Experiences Survey, United States, January–June 2021

Characteristic	%^†^ (95% CI)	p value^§^
**Sex**		**0.29**
Male	34.6 (31.1–38.3)	
Female	36.5 (32.4–40.8)	
**Race and ethnicity**		**0.00**
American Indian or Alaska Native, non-Hispanic	26.7 (18.9–36.3)	
Asian, non-Hispanic^¶,^**	63.9 (54.0–72.7)	
Black, non-Hispanic^¶,^**	55.2 (50.2–60.1)	
Hispanic or Latino^¶^	41.5 (36.2–47.0)	
Multiracial, non-Hispanic^¶,^**	54.5 (44.9–63.8)	
Native Hawaiian or other Pacific Islander, non-Hispanic^¶^	48.5 (32.2–65.2)	
White, non-Hispanic	22.5 (20.0–25.2)	
**Grade^††^**		**0.55**
9	36.8 (32.0–41.9)	
10	34.0 (30.4–37.7)	
11	35.0 (30.7–39.4)	
12	36.6 (31.9–41.5)	
**Poor mental health during the COVID-19 pandemic^§§^**		**0.00**
Ever (always, most of the time, sometimes, rarely)	38.1 (34.6–41.7)	
Never	23.6 (19.9–27.7)	
**Virtual connection with family, friends, or other groups outside of school during the COVID-19 pandemic^§§^**		**0.94**
Never	35.7 (30.4–41.5)	
Ever (always, most of the time, sometimes, rarely)	35.5 (32.1–39.2)	
**Serious difficulty concentrating, remembering, or making decisions because of a physical, mental, or emotional problem^§§^**		**0.00**
Yes	44.1 (39.6–48.7)	
No	28.6 (25.4–32.0)	
**Feel close to persons at your school^§§^**		**0.00**
No (not sure, disagree, strongly disagree)	40.7 (36.9–44.7)	
Yes (strongly agree, agree)	29.6 (25.9–33.6)	
**Total**	**35.6 (32.2–39.2)**	**NA**

**Abbreviation:** NA = not applicable.

* On the basis of the answer (“never” versus “ever” [rarely, sometimes, most of the time, always]) to the survey question, “During your life, how often have you felt that you were treated badly or unfairly in school because of your race or ethnicity?”

^† ^Estimates are weighted.

^§^ Statistical significance defined as p<0.05, by chi-square test.

^¶^ Pairwise *t*-test significantly different from non-Hispanic American Indian or Alaska Native and non-Hispanic White students (p<0.05).

** Pairwise *t*-test significantly different from Hispanic or Latino students (p<0.05).

^††^ No significant pairwise differences (p<0.05).

^§§^ See Table 1 for variable definition.

**TABLE 3:** Percentage of high school students who reported experiencing perceived racism during their life,* by sex, grade, and self-reported race and ethnicity — Adolescent Behaviors and Experiences Survey, United States, January–June 2021

Characteristic	American Indian or Alaska Native, non-Hispanic	Asian, non-Hispanic	Black, non-Hispanic	Hispanic or Latino	Multiracial, non-Hispanic	White, non-Hispanic
	%^†^ (95% CI)	%^†^ (95% CI)	%^†^ (95% CI)	%^†^ (95% CI)	%^†^ (95% CI)	%^†^ (95% CI)
**Sex^§^**						
Male	29.7 (21.9–39.0)	62.5 (54.1–70.1)	52.2 (44.9–59.3)	35.0 (29.7–40.6)	54.5 (44.1–64.5)	25.0 (21.7–28.8)
Female	23.6 (10.8–44.0)	65.7 (48.8–79.4)	58.1 (49.7–66.0)	47.3 (41.4–53.4)	54.7 (44.0–65.0)	19.9 (16.9–23.2)
**Grade^¶^**						
9	—**	57.7 (41.6–72.4)	56.1 (48.5–63.4)	43.8 (36.6–51.2)	45.8 (32.1–60.1)	24.7 (20.6–29.4)
10	—	66.8 (56.4–75.7)	47.9 (37.8–58.2)	36.7 (29.8–44.2)	61.8 (51.6–71.0)	22.0 (18.3–26.3)
11	—	54.3 (35.0–72.4)	56.2 (44.3–67.4)	43.9 (35.3–52.9)	48.5 (36.0–61.1)	21.4 (18.0–25.3)
12	—	75.7 (66.3–83.1)	60.5 (49.7–70.3)	40.9 (34.8–47.4)	58.8 (39.9–75.3)	21.7 (18.0–25.9)
**Total**	**26.7 (18.9–36.3)**	**63.9 (54.0–72.7)**	**55.2 (50.2–60.1)**	**41.5 (36.2–47.0)**	**54.5 (44.9–63.8)**	**22.5 (20.0–25.2)**

* On the basis of the answer (“never” versus “ever” [rarely, sometimes, most of the time, always]) to the survey question, “During your life, how often have you felt that you were treated badly or unfairly in school because of your race or ethnicity?”

^† ^Estimates are weighted.

^§^ Chi-square test indicate significant difference (p<0.05) among the following subgroups of students: Hispanic or Latino males versus females; non-Hispanic White males versus females.

^¶^ No significant pairwise differences in grade across racial and ethnic populations (p>0.05).

** Dashes indicate that results are suppressed because n<30.

**TABLE 4:** Percentage of high school students who reported experiencing perceived racism during their life,* by selected behavioral characteristics and self-reported race and ethnicity — Adolescent Behaviors and Experiences Survey, United States, January–June 2021

Characteristic	American Indian or Alaska Native, non-Hispanic	Asian, non-Hispanic	Black, non-Hispanic	Hispanic or Latino	Multiracial, non-Hispanic	White, non-Hispanic
	%^†^ (95% CI)	%^†^ (95% CI)	%^†^ (95% CI)	%^†^ (95% CI)	%^†^ (95% CI)	%^†^ (95% CI)
**Poor mental health during the COVID-19 pandemic^§,¶^**						
Ever (always, most of the time, sometimes, rarely)	27.6 (16.2–43.0)	67.9 (56.1–77.8)	62.1 (55.4–68.3)	45.7 (38.5–53.1)	55.2 (45.2–64.7)	24.5 (22.1–27.1)
Never	—**	40.5 (30.2–51.7)	38.5 (30.3–47.3)	22.9 (17.4–29.5)	41.7 (26.6–58.4)	12.7 (9.3–17.0)
**Virtual connection with family, friends, or other groups outside of school during the COVID-19 pandemic?^¶,††^**						
Never	—	79.1 (45.4–94.5)	32.2 (23.1–43.0)	41.1 (30.7–52.4)	62.0 (36.2–82.4)	26.1 (19.8–33.6)
Ever (always, most of the time, sometimes, rarely)	31.3 (21.2–43.7)	62.8 (52.7–71.9)	58.4 (52.8–63.7)	41.5 (36.3–46.8)	52.8 (43.6–61.8)	22.6 (19.9–25.5)
**Serious difficulty concentrating, remembering, or making decisions because of a physical, mental, or emotional problem^¶,§§^**						
Yes	39.5 (25.6–55.4)	71.6 (61.3–80.0)	66.9 (59.5–73.5)	52.9 (44.2–61.5)	68.7 (54.3–80.3)	28.4 (25.0–32.1)
No	18.1 (11.7–26.8)	55.9 (43.1–68.0)	47.5 (41.9–53.2)	30.8 (26.1–36.0)	41.2 (31.0–52.1)	18.5 (15.5–21.8)
**Feel close to persons at your school^¶,¶¶^**						
No (not sure, disagree, strongly disagree)	26.6 (17.9–37.7)	68.8 (59.0–77.2)	57.0 (51.0–62.8)	45.3 (38.9–51.9)	62.8 (51.4–72.8)	26.5 (23.5–29.8)
Yes (strongly agree, agree)	31.5 (18.7–48.0)	56.0 (42.4–68.8)	51.7 (43.9–59.3)	36.4 (29.2–44.2)	45.3 (34.9–56.1)	19.4 (16.2–23.1)

* On the basis of the answer (“never” versus “ever” [rarely, sometimes, most of the time, always]) to the survey question, “During your life, how often have you felt that you were treated badly or unfairly in school because of your race or ethnicity?”

^† ^Estimates are weighted.

^§ ^Chi-square test indicates significant difference (p<0.05) among the following subgroups of students: non-Hispanic Asian, non-Hispanic Black, Hispanic or Latino, and non-Hispanic White.

^¶^ See Table 1 for variable definition.

** Dashes indicate that results are suppressed because n<30.

^††^ Chi-square test indicates significant difference (p<0.05) among non-Hispanic Black students.

^§§ ^Chi-square test indicates significant difference (p<0.05) among the following subgroups of students: non-Hispanic Black, Hispanic or Latino, non-Hispanic multiracial, and non-Hispanic White.

^¶¶^ Chi-square test indicates significant difference (p<0.05) among the following subgroups of students: non-Hispanic multiracial and non-Hispanic White.

## Results

During January–June 2021, approximately one third (35.6%) of all high school students reported they were “ever” treated badly or unfairly in school because of their race or ethnicity during their lifetime (i.e., perceived racism). Analyses indicated significant differences in student reports of perceived racism across racial and ethnic populations and behavioral characteristics (Table 2). Perceived racism was highest among Asian students (63.9%), followed by Black (55.2%) and multiracial students (54.5%). Prevalence of perceived racism for Asian, Black, NH/OPI, Hispanic, and multiracial students was higher than perceived racism for White (22.5%) and AI/AN students (26.7%). Higher prevalences of perceived racism were reported among students with poor mental health (38.1% versus 23.6%); those with difficulty concentrating, remembering, or making decisions (44.1% versus 28.6%); and those that did not feel close to persons at their school (40.7% versus 29.6%). When stratified by student report of virtual connection with family, friends, and other groups outside of school, no significant difference in perceived racism was found. When stratified by race and ethnicity, reports of perceived racism varied by sex for Hispanic students (females: 47.3%; males: 35.0%) and White students (males: 25.0%; females: 19.9%), yet patterns were not consistent across groups (Table 3).

Differences in mental health and behavioral characteristics by student report of perceived racism also were observed when stratified by race and ethnicity (Table 4). Perceived racism was higher among students who reported their mental health during the pandemic was not good compared with those with no reported mental health concerns during the pandemic and among Asian (67.9% versus 40.5%), Black (62.1% versus 38.5%), Hispanic (45.7% versus 22.9%), and White students (24.5% versus 12.7%). Perceived racism was lower for Black students who reported not having virtual connection with family, friends, and other groups during the COVID-19 pandemic compared with those who did (32.2% versus 58.4%). Perceived racism was higher among students who reported difficulty concentrating, remembering, or making decisions compared with those who did not: multiracial (68.7% versus 41.2%), Black (66.9% versus 47.5%), Hispanic (52.9% versus 30.8%), and White students (28.4% versus 18.5%). Finally, perceived racism was higher among students who reported they did not feel close to persons at their school compared with those who did: multiracial (62.8% versus 45.3%) and White (26.5% versus 19.4%).

## Discussion

Approximately one in three high school students reported perceived racism during their lifetime, including two thirds of Asian and more than half of Black and multiracial students. Student perceptions of racism were associated with poor mental health; difficulty concentrating, remembering, or making decisions; and a lack of connection with persons at school during the COVID-19 pandemic.

These findings are consistent with other studies on racism and health inequities during the COVID-19 pandemic. The pandemic contributed to increased racism against Asian communities; anti-Asian sentiment (including racist names) stemmed from origination theories of SARS-CoV-2, the virus that causes COVID-19 (*10*). From March 2020 to February 2021, a period covering ABES data collection, COVID-19 hospitalization rates were consistently higher among Black, Hispanic, and AI/AN populations than among White populations (*8*). In addition to COVID-19, events in 2020, such as the killings of Ahmaud Arbery and George Floyd and the Black Lives Matter movement, highlighted the increased racial tension, systemic racism, and structural racism experienced by Black Americans (*11*,*12*).

In contrast, White and AI/AN students had the lowest levels of perceived racism among all student populations. In addition, an association was observed between students’ perceived racism and mental health. All racial and ethnic populations that reported poor mental health also reported a higher prevalence of perceived racism compared with AI/AN students. This finding was unexpected, considering prior research demonstrated widespread personal experiences of discrimination among indigenous populations (*13*).

Although lower than most other racial and ethnic populations, approximately one fourth of White students reported perceived racism. Associations between perceived racism and behavioral characteristics for White students were similar to other racial and ethnic populations. Perceived racism was higher among White students with poor mental health; difficulty concentrating, remembering, or making decisions; and a lack of connection with persons at their school. These findings might be linked to White students who experience status hierarchy threat, described as when racial progress by minority populations is associated with an increase in perception of discrimination against White persons (*14**,**15*).

Finally, the finding that Black students reported less perceived racism when they did not have virtual connection with family, friends, or other groups outside of school is counterintuitive and hard to explain. More research is needed on how the influence of social media and virtual connection with other groups (e.g., friends) outside of school might not be uniformly supportive or health promoting for all students and that the lack thereof might be protective.

## Limitations

General limitations for the ABES are available in the overview report of this supplement, including that causality or directionality of observed associations cannot be determined (*9*). The findings in this report are subject to three specific limitations. First, many of the ABES behavioral questions were asked within the context of the pandemic, and experiences associated with perceived racism might have occurred outside of the period of the pandemic. Because these data are cross-sectional, the extent to which events during the pandemic contributed to lifetime perceived racism at school among students cannot be determined. Second, the single-item, largely interpersonal measure of perceived racism used in this study might not account for the complexity of all racial and ethnic populations’ cultural and structural experiences of racism. In addition, structuring of the response options for several questions might have introduced bias into the study results. Finally, school environment was not accounted for in this analysis. School-level demographic characteristics (e.g., geographic region, racial and ethnic composition of school student body, and socioeconomic status) might have influenced study findings.

## Future Directions

Clear steps to promote awareness of and mitigate racism in schools are critical because of the associations between negative mental health and behavioral characteristics with perceived racism among adolescents. Although there are benefits to school-based antiracism interventions, these actions are rarely implemented in schools because of multiple factors, including political and social variables (*16*). This analysis also points to the importance of a more in-depth examination of the AI/AN student population’s low levels of perceived racism. Lessons learned from this group might be applied to other racial and ethnic student populations to help reduce racism and perceptions of racism. Future quantitative research should include a more in-depth examination of factors moderating associations between perceived racism and health behaviors among adolescents, explore the intersection of perceived racism and behavioral health outcomes for youths with multiple marginalized identities (e.g., sexual minority youths and youths with disabilities), review the intersections of race and ethnicity and sex, and explore longitudinal and cohort studies to understand the causality of racism and poor health outcomes.

## Conclusion

The ABES nationally representative findings demonstrate that at least half of Asian, Black, and multiracial U.S. high school students reported experiencing racism during their life. Notably, perceived racism was reported by students belonging to all racial and ethnic groups, with higher prevalence among students who reported poor mental health during the COVID-19 pandemic, not feeling close to persons at school, and difficulty concentrating, remembering, or making decisions than those who did not report such mental health and behavioral characteristics. Collectively, these findings are similar to other research that describes an association of discrimination and inequity and poor health outcomes (*3*). A better understanding of how negative health outcomes are associated with student experiences of racism can guide training for staff and students to promote cultural awareness and antiracist and inclusivity interventions, which are critical for promoting safe school environments for all students. 
